# Underlying Ossification Phenotype in a Murine Model of Metastatic Synovial Sarcoma

**DOI:** 10.3390/ijms21072636

**Published:** 2020-04-10

**Authors:** Matthew Kirkham, Austen Kalivas, Kaniz Fatema, Sarah Luelling, Brooke H. Dubansky, Benjamin Dubansky, Kevin B. Jones, Jared J. Barrott

**Affiliations:** 1 Department of Biomedical and Pharmaceutical Sciences, Idaho State University, Pocatello, ID 83201, USA; kirkmatt@isu.edu (M.K.); amkalivas@gmail.com (A.K.); fatekan2@isu.edu (K.F.); luelsara@isu.edu (S.L.); 2 Department of Medical Laboratory Sciences & Public Health, Tarleton State University, Fort Worth, TX 76104, USA; dubansky@tarleton.edu; 3 Department of Biological Sciences, University of North Texas, Denton, TX 76203, USA; Benjamin.Dubansky@unt.edu; 4 Departments of Orthopaedics and Oncological Sciences, Huntsman Cancer Institute, University of Utah School of Medicine, Salt Lake City, UT 84112, USA; kevin.jones@hci.utah.edu

**Keywords:** synovial sarcoma, metastasis, heterotopic ossification, bone development genes, inflammation, intralesional calcification, parathyroid hormone-like hormone

## Abstract

Synovial sarcoma, an uncommon cancer, typically affects young adults. Survival rates range from 36% to 76%, decreasing significantly when metastases are present. Synovial sarcomas form in soft tissues, often near bones, with about 10% demonstrating ossification in the tumor. The literature is inconclusive on whether the presence of ossification portends a worse prognosis. To this end, we analyzed our genetic mouse models of synovial sarcoma to determine the extent of ossification in the tumors and its relationship with morbidity. We noted higher ossification within our metastatic mouse model of synovial sarcoma. Not only did we observe ossification within the tumors at a frequency of 7%, but an even higher frequency, 72%, of bone reactivity was detected by radiography. An enrichment of bone development genes was associated with primary tumors, even in the absence of an ossification phenotype. In spite of the ossification being intricately linked with the metastatic model, the presence of ossification was not associated with a faster or worse morbidity in the mice. Our conclusion is that both metastasis and ossification are dependent on time, but that they are independent of one another.

## 1. Introduction

Synovial sarcoma is a soft-tissue malignancy with mesenchymal origins [1,2,3]. These tumors are predominantly found in the extremities, can arise anywhere in the body, and have a predilection for developing adjacent to bone [1,4]. One hypothesis is that the bone provides an anti-apoptotic niche for transformed cells to grow by secreting the decoy receptor osteoprotegerin (OPG) [4]. Furthermore, we traced one cell of origin to a mesenchymal progenitor found in the periosteum [4]. Not only do synovial sarcomas arise next to bone, but ossification can occur within human synovial sarcomas [5,6,7,8,9]. This is a rare event (< 10%) in an already rare cancer type: 3 patients per 1 million [1,3,7,10]. The impact of ossification in synovial sarcoma remains unclear. However, we hypothesized that ossification in synovial sarcoma is correlated with a worse prognosis after observing it in our metastatic mouse model.

To understand the prognostic impact of ossification in synovial sarcoma, we evaluated the prevalence and outcomes in mice with synovial sarcoma, using genetically engineered mice that express either the fusion oncogene *SS18-SSX1* or *SS18-SSX2* [2]. These mice have been studied extensively for their pathophysiology, histology, and molecular expression profiles [2,4,10,11,12,13,14]. Iterations with accompanying genetic manipulations of tumor suppressor genes and oncogenes have been evaluated to approach the most relevant preclinical model. The model that best recapitulates the metastatic biology of human synovial sarcoma is the combined expression of *SS18-SSX1* or *SS18-SSX2* and the deletion of *Pten* via the injection of TATCre, a critical tumor suppressor gene that acts to suppress the over-proliferation of tumor cells [12]. This genetic manipulation is temporally and spatially controlled by the injection of the protein TATCre. In this study, we looked at the prevalence of ossification within genetically engineered mouse models of synovial sarcoma and correlated the survival and tumor progression with the presence of ossification.

## 2. Results

### 2.1. Gross and Histological Analysis of Mouse Synovial Sarcoma Demonstrates Overt Ossification in a Minority of Cases

This study included 463 mice with some variants of synovial sarcoma. The four subcategories were the homozygous expression of SS18-SSX1 (hSS1) (*n* = 88), heterozygous expression of hSS1 (*n* = 94), homozygous expression of SS18-SSX2 (hSS2) (*n* = 124), and heterozygous expression of hSS2 (*n* = 157) (Table 1). *Pten* was concomitantly deleted in all mice using the *Pten^lox5/lox5^* allele, with the deletion of *Pten* occurring spatially via the injection of TATCre in hind limbs in order to drive primary tumor development.

**Figure 1:**
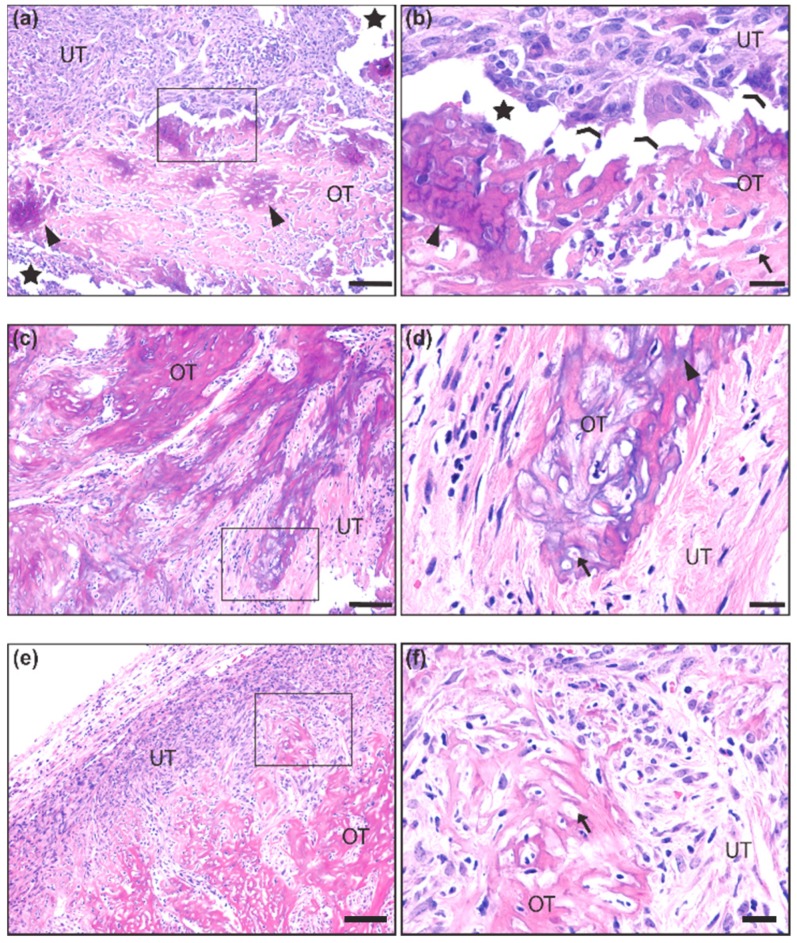
Histological representation of ossification in mouse synovial sarcoma. (**a**,**b**) 10× and 40× insert of synovial sarcoma with ossified tumor matrix (OT) exhibiting calcium salt crystals (purple) embedded in osteoid (pink). The unossified tumor matrix (UT) contains sarcoma cells that are more epithelioid in appearance, and two osteoclast-like multinucleated giant cells are seen at the interface of the ossified matrix (**c**,**d**) 10× and 40× insert of synovial sarcoma with a more extensively calcified matrix interfaced with spindle-shaped tumor cells that are loosely packed in a fibrous stroma. (**e**,**f**) 10× and 40× insert of synovial sarcoma with uncalcified osteoid matrix interfaced with a dense population of spindle-shaped tumor cells embedded in a fibrous stroma. Scale bars in the 10× images = 100 μm, and scale bars in the 40× images = 20 μm. OT = Ossified Tumor, UT = Unossified Tumor, Arrow = lacunae, Arrowhead = calcium salt deposits of ossified tumor matrix, Chevron = osteoclast-like multinucleated giant cells, Star = artificial space at the interface of ossified and unossified tumor matrix.

We first made observations upon tumor resection during necropsy on whether tumors exhibited unusual hardness or bone-like structures. We next analyzed histological slides after hematoxylin and eosin (H&E) staining for the presence of ossification (Figure 1a–f). Ossification was identified by the presence of an eosinophilic (pink) uncalcified boney matrix (i.e., osteoid) (Figure 1a,b,e,f) with some regions exhibiting basophilic (purple) calcium salt deposits (Figure 1a,d). Both calcified and uncalcified regions contained cells embedded in small lacunae, typical of an ossified or ossifying matrix (Figure 1b,d,f). Many lacunae were empty, likely an artifact caused by histological processing. Coalescing trabeculae of the ossified matrix were integrated into the non-ossifying tumor stroma, which consisted of numerous spindle-shaped cells with a mix of small, hyperchromatic nuclei and large, euchromatic nuclei with prominent nucleoli, mitotic bodies, and giant multinucleated cells that resembled osteoclasts (Figure 1b). Out of the 463 mice observed, only 33 (7.1%) demonstrated these ossification characteristics (Figure 1).

### 2.2. Differences in the Ossification Phenotype between Heterozygous and Homozygous Fusion Gene Expression

**Figure 2:**
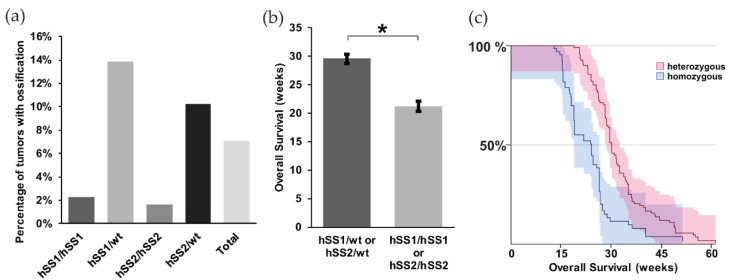
Prevalence of ossification within mouse synovial sarcomas. Representative bar graphs of: (**a**) the percentage of mice showing evidence of ossification among heterozygous (*n* = 251) and homozygous (*n* = 212) phenotypes of *hSS1* and *hSS2*. Only 7.1% of total mice exhibited ossification through palpitation; (**b**) the overall survival of heterozygous and homozygous genotypes in weeks, demonstrating heterozygous phenotypes to have a higher survival rate than homozygous genotypes; and (**c**) the Kaplan Meier Survival curve of heterozygous and homozygous genotypes. Shaded area represents 90% CI. * *p* < 0.05.

When we further looked at the different variants of synovial sarcoma within our model, we noted that the heterozygous expression of either hSS1 or hSS2 had a higher prevalence of ossification compared to the homozygous group, 13.8% and 10.2%, respectively (Figure 2a). This is likely due to the slow development of ossification and a faster disease progression in mice homozygous for either hSS1 or hSS2. As such, mice homozygous for either gene became moribund at 8.3 weeks after TATCre injection, before ossification could occur (Figure 2b,c) (z = 6.54, *p* < 0.001).

### 2.3. Bone Reactivity Detected by Radiography at a Higher Incidence than Histological Ossification

**Figure 3:**
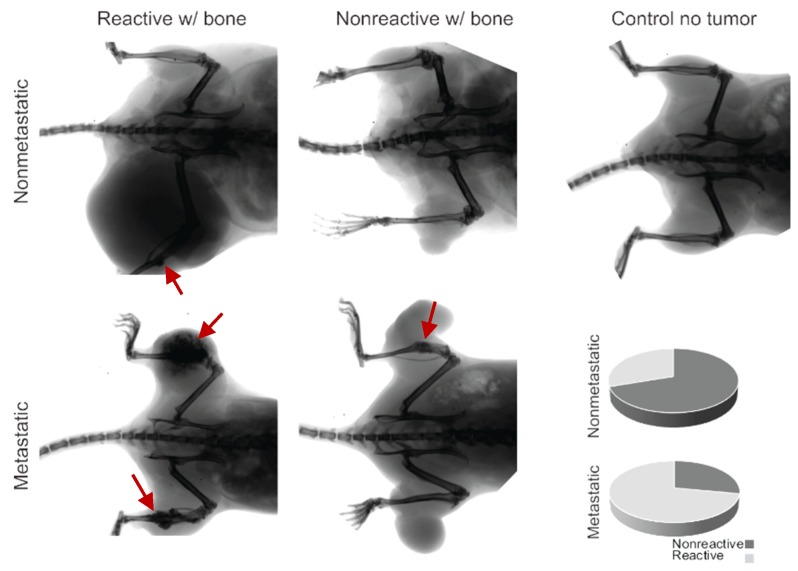
Radiographic imaging of metastatic and nonmetastatic mice with the development of ossifying tumors, showing bone inflammation (red arrows) and interaction or lack of interaction for both groups. The control demonstrates the lack of ossification in the healthy mouse model, and the pie charts indicate the proportionality of reactivity in nonmetastatic mice (30%) and metastatic mice (72%).

We radiographically imaged 50 random synovial sarcomas on 31 mice to assess gross interactions between the bone and the tumor. Some of the mice had bilateral tumors in the hindlimbs. We observed that 72% of the metastatic sarcomas that express heterozygous hSS1 or hSS2 exemplified abnormal bone inflammation or direct growth and interaction at the surface of the bone (Figure 3, Table 2).

The prevalence of bone inflammation in synovial sarcomas in these mice was significantly higher than expected. All mice exhibiting ossification belonged to the hSS1/wt or hSS2/wt subcategory with Pten loss, which is also referred to as our metastatic model of synovial sarcoma. The hSS1/hSS2 heterozygous metastatic categories exhibited a mean 11.6% penetrance of the ossification phenotype upon gross and histological examination (Figure 1 and Figure 2). We further investigated the hSS1/wt or hSS2/wt subcategory with wildtype Pten, also referred to as our nonmetastatic model of synovial sarcoma. It was noted that in the 10 nonmetastatic synovial sarcomas imaged, only three displayed bone interaction; this interaction was also not as extensive and invasive as seen in the metastatic model (Figure 3). Using a Fisher’s Exact Test, we determined that this was significantly lower than the 72% seen in the metastatic model (*p*-value = 0.024) (Table 1).

### 2.4. Underlying Gene Expression of Bone Development Genes in Metastatic Model of Synovial Sarcoma

**Figure 4:**
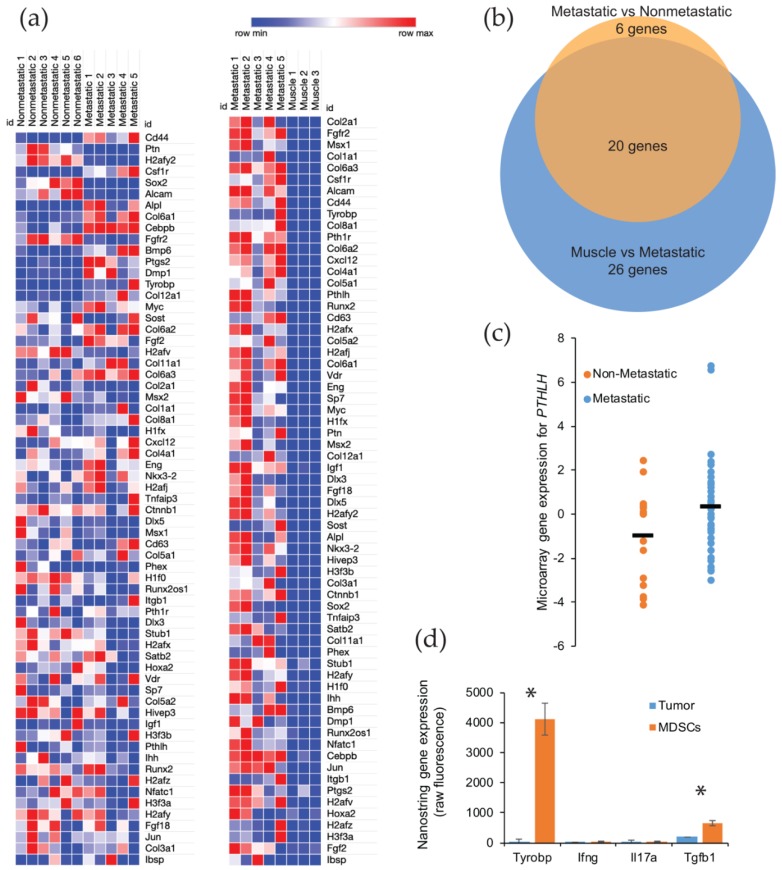
Gene expression of the ossification genes found upregulated in mouse and human metastatic synovial sarcoma. (**a**) Heatmap showing RNA expression and the upregulation (red) or downregulation (blue) of bone development genes in *Pten* loss-induced tumors comparing nonmetastatic to metastatic (left) and metastatic to muscle tissue (right); (**b**) 46 genes were analyzed and determined to be statistically different between metastatic and normal muscle tissue by an adjusted *p*-value threshold of < 0.05. Venn diagram representing statistically significant bone development genes from nonmetastatic vs. metastatic samples (orange, 26 genes) and muscle vs. metastatic samples (blue, 46 genes) showing an overlap of 20 genes of interest between groups; (**c**) Comparison of nonmetastatic to metastatic human synovial sarcoma sample expression of *PTHLH*, showing the spread and mean (bar) of each group. Statistical significance was found with regards to the upregulation of *PTHLH* in metastatic tumors in these patients. *p*-value = 0.039; (**d**) After fluorescence-activated cell sorting, NanoString sequencing was performed to identify the gene expression for secreted proteins involved in ossification that were unique to myeloid-derived suppressor cells (MDSCs). * *p* < 0.05.

We further investigated the relative role of osteogenic signaling in metastatic versus nonmetastatic synovial sarcomas. We analyzed the RNA expression for 65 genes involved in osteoblast differentiation. Only one of the tumors used for these analyses was identified with an ossifying center, though all tumors demonstrated a significant upregulation of genes involved in bone development. This was especially observable when comparing the gene expression in metastatic synovial sarcomas versus control muscle tissue. The enrichment of osteogenic genes of interest was observable for nearly all bone development genes in all sarcoma tissues, with no upregulation of these genes detected in any of the muscle tissues, as would be expected (Figure 4a) (Supplemental Table S1).

After comparing the primary tumors from our metastatic model with muscle, we next evaluated if there was a significant enrichment between the nonmetastatic model and the metastatic model (Supplemental Table S2). Using a *p* value cut off of < 0.05 that was corrected for multiple hypothesis testing, we identified 46 genes that were significantly upregulated between the normal muscle tissue and the primary synovial sarcomas from the metastatic model. A similar comparison of gene enrichment was conducted between primary tumors from nonmetastatic and metastatic mice, and we detected 26 bone development genes that were upregulated in the tumors from the metastatic model. Not surprisingly for these upregulated genes, 20 genes were shared between the two comparisons (Figure 4b) (Supplemental Table S3). The six unshared genes (CEBPB, BMP6, PTGS2, DMP1, FGF2, and H2AFV) are likely the important contributors to the ossifying phenotype seen in the metastatic synovial sarcomas.

To further explore the relationship in upregulated genes in the metastatic mouse model, we turned our attention to a human database. Using the NCBI GEO Dataset GSE54187, human synovial sarcomas were bifurcated into nonmetastatic (*n* = 15) and metastatic (*n* = 42). The 47 genes identified in the mouse comparison were analyzed across the human tumors, and only one gene was found to be significantly different: parathyroid hormone-like hormone (PTHLH) (*p*-value = 0.0391, 95% CI = −2.56 to −0.068) (Figure 4c) (Supplemental Table S3). This gene is intriguing in that it is downstream of RUNX2, a master regulator of bone development and ossification, and a high expression has been correlated to a faster disease progression in a number of cancers (i.e., colon, prostate, breast, head, and neck) due to the overexpression of PTHLH [15,16,17,18]. Among its putative functions for cancer progression is the activation of myeloid-derived suppressor cells (MDSCs) in the bone marrow [18]. These immune cells have the ability to promote growth and angiogenesis, while interfering with T cell immune responses [19]. We posited that these recruited MDSCs could also be triggering an ossification phenotype in our metastatic model of synovial sarcoma. We have already seen an overwhelming presence of macrophages, monocytes, and neutrophils within the tumor microenvironment of metastatic synovial sarcomas in mice [12]. We measured signals from the sorted populations by NanoString sequencing and found several genes that were uniquely expressed in the MDSCs and have a functional history in ossification (Figure 4d). The gene that represented the largest enrichment in MDSCs (130-fold) was Tyrobp (*p*-value = 0.0006). This gene is involved in bone cyst formation in Nasu-Hakola disease [20]. Other genes that were identified as potential sources of ossification were Ifng, Il17, and Tgfb1 [21]. All of these exhibited a 4-fold enrichment over the tumor cells, but only Tgfb1 was statistically significant (*p*-value = 0.0076) (Supplemental Table S4).

### 2.5. Gross Metastasis is Loosely Correlated with the Presence of Ossification, but Survival is More Favorable in Mice with Ossification

We noticed a trend among mice with synovial sarcomas and intratumoral ossification: they exhibited an increased prevalence of gross metastasis. Within the heterozygous mice, for either hSS1 or hSS2, we already noted a higher prevalence of ossification and an associated longer survival in heterozygous mice. When we compared the fraction of metastatic mice between sarcomas with and without ossification, we observed that gross metastasis increased from 42% to 65% in synovial sarcomas with ossification. However, the statistical analysis by Fisher’s Exact Test only demonstrated a *p*-value of 0.086 (Table 3).

**Figure 5:**
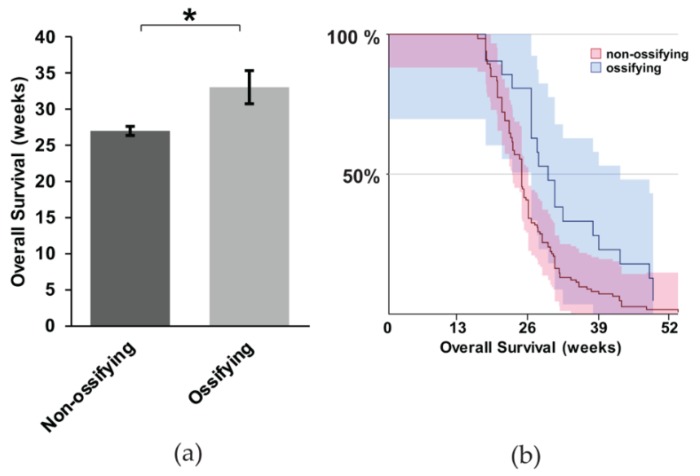
The correlation between ossification and mouse survival with metastatic synovial sarcoma; (**a**) Representative bar graph showing the overall survival of non-ossifying (*n* = 104) and ossifying (*n* = 20) mice, with statistical significance occurring for the survival of mice exhibiting ossification; (**b**) Kaplan Meier Survival curve of non-ossifying and ossifying mice. Shaded area represents 90% CI. * *p* < 0.05.

It is unclear if ossification portends a worse prognosis in synovial sarcoma. In particular, considering the evidence that metastasis is more obviously present when synovial sarcomas exhibit ossification, we wanted to investigate this correlation more thoroughly. We performed a Kaplan Meier Survival analysis between mice with and without ossification. We used the heterozygous mice expressing hSS1 or hSS2 for the analysis. The mice with ossification exhibited, on average, a six-week longer survival (Figure 5a,b) (z = 2.81, *p* = 0.0005). This contradicts our hypothesis that ossification might contribute to metastasis and a faster time to morbidity, at which point mice were euthanized per protocol to prevent unnecessary suffering, thus not achieving full mortality.

**Figure 6:**
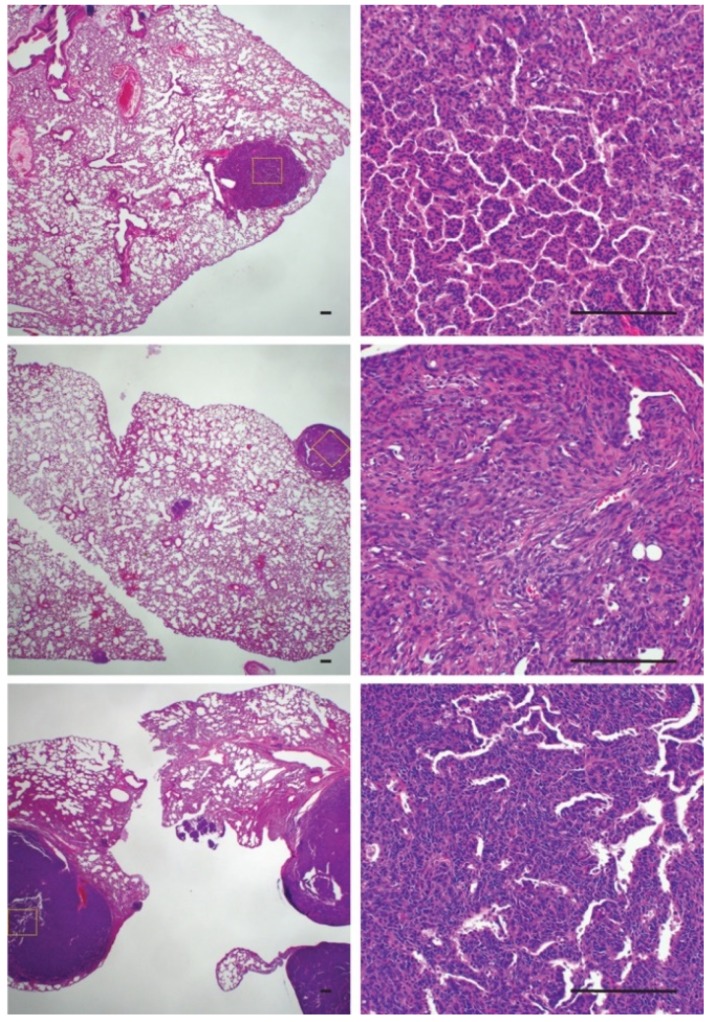
Histological sections of pulmonary metastases demonstrating a lack of ossification within tumors. Scale bars = 100 μm.

The increased rate of metastasis in ossifying synovial sarcomas raised questions about the cellular phenotype of the metastatic lesions. To investigate if ossified cells or cells with the potential to ossify were more likely to metastasize and colonize the lungs, we evaluated pulmonary synovial sarcoma metastases for the presence of ossification (Figure 6). Only one mouse that exhibited rampant metastatic disease with large pulmonary metastases displayed tumors that were solid upon palpitation. All the other mice with lung metastases demonstrated a normal synovial sarcoma morphology by H & E, with no obvious presence of ossification (Figure 6). This led us to conclude that metastasis and ossification are independent events but that both are dependent on time.

## 3. Discussion

Prior to our investigation, there was uncertainty about the ossification phenotype observed in synovial sarcoma and its correlation to outcome [5,6,8,22]. Using a mouse model of synovial sarcoma that exhibits an overall penetrance of 7.2% ossification, we examined the correlation of ossification with pulmonary metastasis and outcome. While we did see more ossification within our metastatic model of synovial sarcoma than for the nonmetastatic model, we demonstrated that a nonsignificant trend in mice with ossifying primary tumors showed gross metastatic lesions in the lungs upon necropsy. However, the evaluation of survival was more favorable for mice with detectable ossification. This led us to conclude that both the development of pulmonary metastasis and ossifying centers in the primary tumor are independent from each other but that both are dependent on time.

Synovial sarcomas were initially thought to arise from muscle precursors [2] but have been shown to originate more readily within the periosteum, the highly vascularized connective tissue that surrounds bone [4]. Though it is a phenotype with a low penetrance and which is not readily apparent, most synovial sarcomas express genes involved in the development of bone [23]. As examined above, ossification and metastasis were not immediately correlated with a worse prognosis, but this can likely be attributed to the morbidity of primary tumor growth necessitating humane euthanasia in faster growing tumors before significant ossification could occur.

Also, worth noting is the consideration that, as observed in Figure 2b, mice heterozygous for *hSS1* or *hSS2* showed a longer survival period than homozygous mice. Having a heterozygous genotype may lead to a greater amount of differentiation within the tumor environment, translating into a better overall survival or prognosis. A higher degree of differentiation often relates to less migration and an easier identification of the original tumor source [24].

An interesting element was the presence of ossification genes, even in the absence of an osteoid matrix and calcium crystal deposits in the primary tumors. While it is apparent that bone development genes from our list of interest are responsible for ossification within synovial sarcoma, it is less clear what factors are driving these bone development genes to be upregulated. One possible consideration is inflammation within the tumor environment. Such inflammation incites an immune response, galvanizing immune cells such as lymphocytes, macrophages, and neutrophils to infiltrate the primary site and secrete growth signaling molecules that are responsible for transforming mesenchymal stem cells into differentiated osteoblast cells [25,26].

*Pten* silencing acts as a major step in promoting and maintaining baseline inflammation through PI3K/AKT signal transduction, resulting in the recruitment of macrophages and neutrophils to the tumor microenvironment [27]. The accumulation of these immune cells leads to the deactivation of T-cells and natural killer cells, which acts to limit cancer cell elimination while simultaneously secreting growth factors such as VEGF and TGF-α, thus promoting angiogenesis and facilitating the endothelial-mesenchymal transition (EndMT) [28,29]. Both of these features act to promote metastasis, while also simultaneously triggering bone reactivity and development. This corresponds well to the patterns of ossification observed in our mouse tumor samples

The most interesting finding was perhaps the *PTHLH* upregulation within the sequencing results among human patients between nonmetastatic and metastatic groups. *PTHLH* aids in regulating cell differentiation and proliferation, particularly in bone development, and promotes cell migration and invasion [16,17]. A significant prevention of apoptosis has also been observed with *PTHLH* upregulation [15]. *RUNX2* (runt-related transcription factor 2), another gene involved in osteoblast differentiation, may stimulate *PTHLH* expression through *IHH* (Indian Hedgehog) [15], both of whose expressions are upregulated in our metastatic model when compared to the muscle model (Figure 4a). The increased expression of *RUNX2*, as seen above, correlates with an increase in *PTHLH* expression. Coupled with the upregulation of *PTH1R* (parathyroid hormone type 1 receptor) seen in our mouse models, *PTHLH* likely plays a significant role in cancer cell proliferation, migration, and invasion [15].

While thoroughly discussed here in the context of synovial sarcoma, soft tissue ossification is not unique to neoplastic disease. Indeed, there are a number of non-neoplastic, progressive, inherited, and acquired disorders of inappropriate bone formation in soft tissues, collectively called heterotopic ossification (HO). Several lab-generated mouse models for both ossifying sarcomas (such as those used in the current study) and HO disorders, have been developed to provide insights into the mechanistic beginnings of HO. Recently, the American alligator (*Alligator mississippiensis*) has been established as a natural animal model for HO because of the development of ectopic bone in some scales [30]. Osteoderm formation shares histologic and, presumably, mechanistic similarities to HO. The development of a boney matrix within the mature dermis initiates approximately 9–12 months post-hatch, when mesenchymal stem cells differentiate into osteoblasts to form qualitatively normal bone [30]. Most forms of HO are triggered and exacerbated by trauma, when faulty mechanisms of inflammation initiate the aberrant cell differentiation of mesenchymal stem cells involved in tissue regeneration during wound healing [31]. Consequently, a local osteogenic program is expressed and leads to the production of heterotopic bone in the affected tissue. The initiating and driving factors for ossification in both osteoderms and HO are the subject of ongoing investigations.

Ossifying synovial sarcomas might undergo an ossification process that is similar to HO lesions, as indicated by the presence of a histologically typical bone matrix, the expression by tumor cells of osteogenic genes, and the contribution of the inflammatory tumor environment to the ossification phenotype. As such, the American alligator may also become an appropriate model for soft tissue ossification in neoplastic disease as well as for HO disorders. Future work to describe the initiation, formation, and prognosis of synovial sarcoma along a protracted developmental time scale will be needed to identify the link between aberrant cell fate mechanisms and tissue environment cues that initiate bone formation in synovial sarcoma. A comparative model between HO and synovial sarcoma could provide a platform to deconstruct conserved components and identify common therapeutic targets for the treatment of both disease states.

## 4. Materials and Methods

### 4.1. Mice

Mouse experiments were conducted with the approval of the University of Utah’s Institutional Animal Care Committee in accordance with legal and ethical standards established by the National Research Council and published in the Guide for the Care and Use of Laboratory Animals (protocol # 14-01016). The previously described Rosa26-LSL-SS18-SSX1;Pten^fl/fl^ and Rosa26-LSL-SS18-SSX2;Pten^fl/fl^ mice were maintained on a mixed strain background, C57BL/6 and SvJ. Mice were genotyped with the following primers: Rosa26-LSL-SS18-SSX (F flox—AAACCGCGAAGAGTTTGTCCTC, F wt—GTTATCAGTAAGGGAGCTGCAGTGG, R—GGCGGATCACAAGCAATAATAACC) Pten (F flox—CAAGCACTCTGCGAACTGAG, R—AAGTTTTTGAAGGCAAGATGC). TATCre was dosed by 10 μL intramuscular injections at 50 μM at 1 month of age.

### 4.2. Histology

Mouse tissues were fixed in 4% paraformaldehyde overnight and embedded in paraffin. Paraffin-embedded tissues were stained by immunohistochemistry by rehydrating slides through a citrosolv and ethanol dilution wash. Hematoxylin and eosin staining was performed as previously described [12].

### 4.3. Transcriptome Analyses

Total RNA was isolated from murine synovial sarcomas and from normal muscle taken from the Sartorius and Rectus femoris with the RNeasy mini kit (QIAGEN, Germantown, MD, USA). For the transcriptome sequencing of TATCre tumors, RNA was prepared using the Illumina TruSeq RNA kit (Illumina, San Diego, CA, USA), checked with the Bioanalyzer RNA 6000 chip (Agilent Technologies), captured using the RiboZero method (Illumina, San Diego, CA, USA), and 50-cycle end-read sequenced on an Illumina HiSeq 2000. Reference fasta files were generated by combining the chromosome sequences from mm10 with splice junction sequences generated by USeq (v8.8.8, Source Forge, Salt Lake City, UT, USA) MakeTranscriptome using Ensembl transcript models (build 74). Reads were aligned with Novoalign (v2.08.01, Novocraft, Selangor, Malaysia), allowing up to 50 alignments per read. USeq’s SamTranscriptomeParser selected the best alignment for each and converted the coordinates of reads aligning to splices back to genomic space. The differential gene expression was measured using USeq DefinedRegionDifferentialSeq, which counts the number of reads aligned to each gene and then calls DESeq2 (v1.4.5, Bioconductor) using default settings.

For the NanoString gene expression analysis, RNA was isolated from three different tumor cell populations taken from Rosa26-LSL-SS18-SSX2;Pten^fl/fl^: GFP+, CD11b+/Ly6C+/Ly6G+, and CD11b+/Ly6Cmid/F4-80+/MHC IIhigh as previously described [12]. In brief, specimens were minced, enzymatically digested (Tumor Dissociation kit; Miltenyi Biotec, San Diego, CA, USA), and mechanically dissociated (GentleMACS Tissue Homogenizer; Miltenyi Biotec, San Diego, CA, USA). Tissue debris was removed using 70-µm MACS Smart strainers (Miltenyi Biotec, San Diego, CA, USA). Cells from all tissues were washed with PBS containing 0.5 mg/mL bovine serum albumin, and then stained using a cocktail of rat anti–mouse antibodies from BD: Ly6C, CD11b, I-A/I-E, F4/80, Ly-6G, and DAPI (Invitrogen, Carlsbad, CA, USA). The cell expression was determined by multicolor flow cytometry on a FACSCanto flow cytometer (BD, San Jose, CA, USA), and cell sorting was performed using a FACSAria (BD, San Jose, CA, USA) and FlowJo 8.7.1 (FlowJo, Ashland, OR, USA) for analysis. 10 ng of RNA was combined with the mouse immunology panel of 545 gene probes and analyzed on the nCounter (NanoString, Seattle, WA, USA).

### 4.4. Statistics and Analysis

Genes of interest were selected from a list of bone development genes that were then verified to have bone expression using https://www.genecards.org/. After confirming the gene, the expression was checked under the TISSUES section. Any genes that had expression in bone or bone marrow were selected as genes of interest.

The *Pten* loss-induced tumor group, our metastatic model (*n* = 5), and the muscle tissue group (*n* = 3) were sampled, and the tissues were processed to determine the gene expression. Fragments per Kilobase of transcript per Million mapped reads (FPKM), Log2, and Adjusted *p*-values were obtained from analysis, and the results were ordered from highest to lowest adjusted *p*-value. Using these FPKM values, a heatmap was generated for all genes of interest.

Shared genes of interest between metastatic vs. muscle and metastatic vs. nonmetastatic tissues were found by sorting columns alphabetically and finding the overlap and were then plotted using Adobe Illustrator in an area-proportional Venn diagram showing the overlap of genes shared between both groups.

Human tumor sample data sets were accessed using NCBI GEO, Series GSE54187, from the Stanford University Department of Pathology. The gene expression data was extracted and analyzed using an unpaired t-test to determine if a statistical significance between metastatic and nonmetastatic tumor samples existed for each gene of interest.

The NanoString data was analyzed for significance by averaging the fluorescence signal from the tumor cells (*n* = 4) and MDSC cells (neutrophils, macrophages, monocytes, *n* = 8) for ossifying genes and performing a one-way ANOVA to determine the statistical significance.

## 5. Conclusions

In the present study, we evaluated the impact of ossification on survival outcome in mice with metastatic synovial sarcoma. In a controlled comparison of mice with metastatic synovial sarcoma and only the presence or absence of ossification as the differentiating phenotype, we observed that mice with ossification exhibited a 6-week longer survival. An underlying gene expression for bone differentiation and inflammation was associated with our metastatic and ossifying phenotypes.

## Figures and Tables

**Table 1:** Genetic variants of the murine model for synovial sarcoma.

	Ossification Phenotype	N
*Rosa-LSL-SS18-SSX1/wt; Pten^fl/fl^*	++	94
*Rosa-LSL-SS18-SSX1/SS18-SSX1; Pten^fl/fl^*	+	88
*Rosa-LSL-SS18-SSX2/wt; Pten^fl/fl^*	++	157
*Rosa-LSL-SS18-SSX2/SS18-SSX2; Pten^fl/fl^*	+	124

**Table 2:** Fisher’s Exact Test comparing the number of tumors with bone interaction between nonmetastatic and metastatic synovial sarcomas.

	Non-Reactive with Bone	Reactive with Bone
Nonmetastatic synovial sarcoma	7	3
Metastatic synovial sarcoma	14	36

**Table 3:** Fisher’s Exact Test comparing metastasis between non-ossifying and ossifying synovial sarcomas.

	Nonmetastatic	Metastatic
Non-ossifying synovial sarcoma	60	44
Ossifying synovial sarcoma	7	13

## Supplementary Materials

Supplementary materials can be found at https://www.mdpi.com/1422-0067/21/7/2636/s1. Supplemental Table S1. RNAseq FPKM values for tumor and muscle. Supplemental Table S2. Gene lists used in Venn diagram. Supplemental Table S3. Raw microarray values for PTHLH expression in human synovial sarcoma. Supplemental Table S4. Nanostring expression data for cell sorted populations.

## Abbreviations

SS	Synovial Sarcoma
OPG	Osteoprotegerin
Pten	Phosphatase and tensin homolog
HO	Heterotopic Ossification
CEBPB	CCAAT/enhancer binding protein
BMP6	Bone morphogenic protein 6
PTGS2	Prostaglandin-endoperoxide synthase 2
DMP1	Dentin matrix protein 1
FGF2	Fibroblast growth factor 2
H2AFV	H2A.Z Variant Histone 2
PTHLH	Parathyroid hormone-like hormone
MDSC	Myeloid derived suppressor cells
RUNX2	Runt-related transcription factor 2
Tyrobp	Transmembrane Immune Signaling Adaptor
Ifng	Interferon gamma
Il17	Interleukin 17
Tgfb	Transforming growth factor beta
PI3K	Phosphoinositide 3-kinase
AKT	Serine/Threonine Kinase
VEGF	Vascular endothelial growth factor
TGF-a	Transforming growth factor alpha
IHH	Indian Hedgehog
PTH1R	Parathyroid hormone 1 receptor
CI	Confidence interval
FPKM	Fragments Per Kilobase of transcript per Million mapped reads
